# 

**Published:** 1845-09

**Authors:** 


### CONTENTS

OF NO. III.

---

### ORIGINAL COMMUNICATIONS.

ESSAYS AND CASES.

ART. I.—Lectures on Diseases of the Chest. By J. R. BUCK, M.D., Lecturer on the Theory aud Practice of Medicine in the Louisville Summer School of Medicine. - - 185

ART. II.—Remarks on the Causes, Symptoms, and Treatment of Diarrhœa, as it appears at Wetumpka, Alabama. By JAMES C. HARRIS, M.D. - - - - - - - - 191

ART. III.—An Account of an Epidemic which prevailed in Henderson county, Kentucky, during the Winter and Spring of 1845. By Dr. A. J. MORRISON, of Henderson, Kentucky. - - - - - - - - - - 198

REVIEWS.

ART. IV.—Lectures on the Theory and Practice of Surgery. By the late ABRAHAM COLLES, M. D. For thirty-four years Professor of Surgery in the Royal College of Surgeons in Ireland. Edited by SIMON M'COY, Esq., F.R. C.S.I. Philadelphia: Ed. Barrington & Geo. D. Haswell. 1845. 8vo. pp. 420. - - - - - - - - - 211

ART. V.—Vestiges of the Natural History of Creation. Second edition, from the third London edition, greatly amended by the author, and an iutroduction, by Rev. GEORGE B. CHEEVER, D.D. N. York: Wiley & Putnam. 1845. 12mo. pp. 280. - - - - - - - - 233

SELECTIONS FROM AMERICAN AND FOREIGN JOURNALS

Gangrene of the Mouth in Children. - - - - - - 245

Analysis of an 'Official Report to the Surgeon-General U. S. Army, on the Use of large Doses of Sulphate of Quinine in Diseases of the South. By JOHN BYRNE, M.D., Assistant Surgeon U. S. Army. - - - - - - - 246
Case of Gastrotomy—Read before the Tennessee State Medical Society, by Dr. I. E. MANLOVE, and forwarded, by vote of the Society, for publication in the Boston Medical and Surgical Journal. - - - - - - - - - - 250
Tumor on the Thigh, affecting the Arterial System. Reported by E. L. DUDLEY, M.D. - - - - - - - - 254
Mesmeric Infirmary. - - - - - - - - - - 255
A Case in which Calomel excited the symptoms of Poisoning by Corrosive Sublimate. - - - - - - - - 256
Fossil Remains. - - - - - - - - - - - 256
Case of Third Dentition. - - - - - - - - 257
Case of Triplets. - - - - - - - - - - - 258
Case of Poisoning by Prussic Acid. - - - - - - 258
Case of Insanity cured by a Surgical Operation. - - - 259
Double Consciousness. - - - - - - - - - - 261
Occupation in Insanity. - - - - - - - - - 262
Ipecacuanha in Emetic Doses, as a powerful restorative in some cases of Exhaustion and Sinking. By JOHN HIGGINBOTHAM, F.R.C.S., Nottingham. (Read before the Nottingham Medico-Chirurgical Society, May 23, 1845.) - 263
French and English opinions on the Treatment of Typhus. - 264
A Case of Abscess in the Groin attended with symptoms of Hernia, from which two Lumbrici Teretes and afterwards Fæcal Matter were discharged, and the patient recovered. By THOMAS HOWELL, Esq., of Risborough. - 265
Death from Hepatic Abscess bursting into the Pericardium. - 267
Ulceration of the Rectum. - - - - - - - - 268

### ORIGINAL INTELLIGENCE.

Vinegar in cases of Narcotic Poisoning. - - - - - - 269
Spontaneous Cure of Consumption. - - - - - - - 270
Remarkable Deformity of a Human Fœtus. - - - - - 271
American Journal of Science and Art. - - - - - - 272
New Medical Journals. - - - - - - - - - 273
Fownes's Chemistry for Students. - - - - - - - 274
Boston Medical and Surgical Journal. - - - - - - 274
Salacine as a substitute for Quinine. - - - - - - - 275
Medical Department of Harvard University. - - - - - 275
Louisville Medical Institute. - - - - - - - - 276
Louisville Summer School of Medicine. - - - - - - 276

